# New Screening Tool for Aortic Root Dilation in Children with Marfan Syndrome and Marfan-Like Disorders

**DOI:** 10.1007/s00246-020-02307-0

**Published:** 2020-01-31

**Authors:** Lidia Wozniak-Mielczarek, Robert Sabiniewicz, Radosław Nowak, Natasza Gilis-Malinowska, Michalina Osowicka, Maksymilian Mielczarek

**Affiliations:** 1 Department of Pediatric Cardiology and Congenital Heart Diseases, Medical University of Gdansk, 7 Debinki Street, 80-211, Gdansk, Poland; 2 2nd Department of Cardiology, Medical University of Gdansk, Gdansk, Poland; 3 1st Department of Cardiology, Medical University of Gdansk, Gdansk, Poland

**Keywords:** Marfan syndrome, Connective tissue diseases, Dilation, Aortic root ratio, Screening

## Abstract

One of the roles of a pediatric cardiologist who suspects or diagnoses a genetically determined connective tissue disease (e.g., Marfan, Ehlers–Danlos, and Loeys–Dietz syndromes) is to assess whether the aortic root is dilated. The aortic root diameter is affected by the patient’s age, sex, and body surface area. Therefore, the aortic root diameter needs to be determined and expressed as a *z*-score. Calculation of the *z*-score is time-consuming and problematic if used infrequently. This study aimed to introduce a simple screening method for identifying aortic root dilation in children. The study population consisted of 190 children who were diagnosed with Marfan syndrome or Marfan-like disorders. The aortic root ratio (ARr) was formulated. The value of the ARr was compared in each patient with the results in *z*-scores, which were obtained using on-line calculators based on the most widespread nomograms. The optimal cut-off value of the ARr was ≥ 18.7. At this cut-off point, the sensitivity of the ARr ranged from 88.3% to 100% and the specificity ranged from 94% to 97.8%. All of the patients in whom the ARr failed to identify aortic root dilation were also divergently classified by different nomograms. At the ARr cut-off point of ≥ 18.0, a sensitivity of 100% was achieved for all nomograms with minimal reduction in specificity. The ARr allows for rapid and precise screening for aortic root dilation in children. Unlike classic analysis, the ARr does not require nomograms or on-line calculations.

## Introduction

Marfan syndrome (MFS) is a systemic disorder of connective tissue caused by mutations in the fibrillin-1 (FBN1) gene [1, 2]. The clinical spectrum of MFS is highly variable, and mainly involves the skeletal, ocular, and cardiovascular systems [3–5]. Cardiovascular involvement in the form of aortic aneurysms and aortic dissections are the leading cause of mortality among patients with MFS [6, 7]. While molecular testing for FBN1 mutations is available, the diagnosis of MFS is still difficult and cannot be established solely with demonstration of a gene mutation [8–10]. Diagnosis of MFS is based on defined clinical criteria called the revised Ghent nosology, where the cardinal feature is aortic root dilation [11]. Similarly, in other genetic connective tissue disorders, such as Ehlers–Danlos or Loeys–Dietz syndrome, the fundamental role of a consulting cardiologist is to determine whether the aortic root is dilated.

The aortic diameter is affected by the patient’s age, sex, and body surface area (BSA). Therefore, establishing a single normal range for the aortic diameter for the entire population is not possible. To determine whether the aortic root is dilated, the aortic root diameter should be assessed by using nomograms and expressing it as a *z*-score adjusted for BSA, age, and sex [12–14]. The *z*-score describes how many standard deviations the value is above or below the mean predicted diameter for the examined patient. Aortic dilation is confirmed when the *z*-score is ≥ 2, which corresponds to an aortic root diameter above the upper limit of the 95% confidence interval of the distribution in a large reference population [11, 12].

In the pediatric population, the most widespread *z*-score formulas are based on nomograms by Gautier et al., Pettersen et al., and Cantinotti et al. [15–17]. The *z*-score formulas are complicated, but they can be calculated using on-line calculators. However, these calculations are time-consuming and can be problematic for professionals who use them infrequently. Therefore, this study aimed to introduce a simple screening method for identifying aortic root dilation in children.

## Material and Methods

### Study Design

We performed a retrospective analysis of 193 children with MFS or Marfan-like disorders who were diagnosed in our department between January 2014 and May 2018. During this time-span, all of the patients underwent at least one transthoracic echocardiography examination with detailed assessment of the ascending aorta. We aimed to create a simple screening method for identification of aortic root dilation. For this purpose, we formulated an aortic root diameter to height ratio and called it the aortic root ratio (ARr). Subsequently, we compared the value of the ARr in each patient with the results in *z*-scores, which were obtained using on-line calculators based on the most widespread nomograms, as described below. Patients who had surgery on the ascending aorta were excluded from the study (3 patients). The research was approved by the local Ethics Committee.

### Study Population

The final study population consisted of 190 children with MFS or Marfan-like disorders, without previous cardiac surgery on the ascending aorta. The study population comprised patients with MFS (*n* = 55), Ehlers–Danlos syndrome (EDS, *n* = 24), Loeys–Dietz syndrome (*n* = 7), ectopia lentis syndrome (*n* = 2), neonatal Marfan syndrome (*n* = 2), MASS phenotype (*n* = 1), and marfanoid habitus (*n* = 99). Among 24 patients with EDS, 11 were diagnosed with hypermobile EDS (type 3), three with classical EDS (type 1), and three with vascular EDS (type 4), and in seven patients, the type of EDS was not specified. Marfanoid habitus was defined as a constellation of symptoms that are characteristic for patients with MFS, including wrist and thumb signs, chest deformities, a reduced upper to lower body segment ratio, increased arm span to height ratio, skin striae, hindfoot deformity, flat foot, scoliosis, thoracolumbar kyphosis, reduced elbow extension, facial features (dolichocephaly, downward slanting palpebral fissures, enophthalmos, retrognathia, and malar hypoplasia), and severe myopia. All of these were included in the systemic score according to the Ghent criteria [11]. We classified patients as having marfanoid habitus once they accrued at least 7 points in the systemic score, but did not meet the Ghent criteria. The mean age of patients was 12.30 ± 4.56 years (range 3 months–18 years) and 88 (46.32%) were female. Detailed information about the patients enrolled into the study is shown in Tables 1 and 2.

**Table 1:** Number of patients enrolled in the study divided according to whether they had Marfan syndrome or Marfan-like disorders

	Number of patients	Excluded^a^	Final number of patients enrolled in the study
Marfan syndrome	57	2	55
Ehlers–Danlos syndrome	24	0	24
Loeys–Dietz syndrome	8	1	7
Ectopia lentis syndrome	2	0	2
Neonatal Marfan syndrome	2	0	2
MASS phenotype	1	0	1
Marfanoid habitus	99	0	99
Total	193	3	190

^a^Patients were excluded because of previous cardiac surgery on the ascending aorta

**Table 2:** Physical features of patients included in the study

	Range	Mean value ± SD	Percentiles, range (mean ± SD)
Age (years)	0.25 (3 months) to 18	12.30 ± 4.56	–
Height (cm)	73–206	160.25 ± 28.90	2–99.9 (78.80 ± 25.30)
Weight (kg)	5.5–86	45.26 ± 18.01	0.1–99.9 (44.78 ± 28.82)
BSA (m^2^)	0.34–2.15	1.44 ± 0.42	–

### Echocardiography

**Fig. 1:**
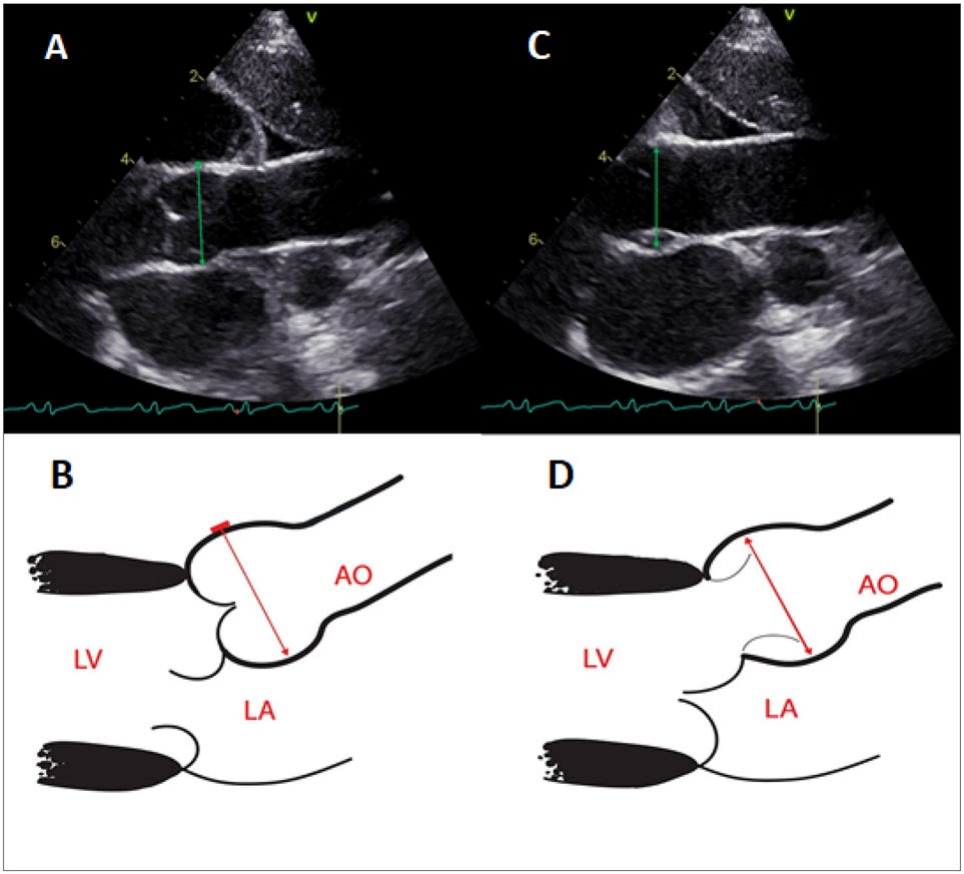
Overview of applied techniques for determining the dimension of the aortic root. **a**, **b** Leading edge in end-diastole. **c**, **d** Inner edge in mid-systole

Transthoracic echocardiography was performed using the Vivid E95 and Vivid S6 ultrasound systems and M5Sc or 6S transducers manufactured by General Electric, Boston, MA. Each echocardiogram was conducted by an experienced cardiologist in accordance with the recommendations of the European Association of Cardiovascular Imaging (EACVI) and the American Society of Echocardiography (ASE) [12]. The aortic root was shown using two-dimensional echocardiography in the parasternal long-axis view. The maximum dimension of the aortic root was measured in millimeters at the sinuses of Valsalva, perpendicular to the long axis of the aorta. Both the above-mentioned documents (EACVI and ASE) recommend the leading edge in end-diastole technique for aortic root measurements. However, in the child population, no uniform method has been established to date for aortic root measurement and there are different nomograms. Therefore, in this study, we performed aortic root measurements using two different techniques: the leading edge in end-diastole and inner edge in mid-systole (Fig. 1a–d).

### Assessment of Aortic Root Dilation

Aortic root dimensions were expressed as *z*-scores. In each patient, the *z*-score was calculated using three different on-line calculators on the basis of nomograms by Gautier et al., Pettersen et al., and Cantinotti et al. These nomograms differ with regard to the applied technique of aortic root diameter measurement. The formula by Gautier et al. corrects the aortic root diameter not only with regard to BSA, but also sex (Table 3). Aortic root dilation was recognized when the *z*-score was ≥ 2.

**Table 3:** Nomograms that were used for *z*-score calculations

Applied nomograms	Measurement technique	Correcting factors
Gautier et al.	Leading edge to leading edge in end-diastole	BSA, sex
Pettersen et al.	Inner edge to inner edge in mid-systole	BSA
Cantinotti et al.	Inner edge to inner edge in mid-systole	BSA

At the same time, the ARr was calculated according to the following formula: aortic root diameter (mm) divided by the patient’s height (cm) multiplied by 100.

Because of the lack of a uniform aortic root measurement technique in the pediatric population, we calculated two variants of the ARr using aortic root dimensions that were measured with two techniques, namely leading edge in end-diastole and inner edge in mid-systole. The obtained results of the ARr were then compared with the *z*-score values that were obtained using formulas based on all three standard nomograms (Gautier et al., Pettersen et al., and Cantinotti et al.).

### Statistical Analysis

Continuous data are presented as the mean value and standard deviation (SD) or as median and interquartile range. Categorical data are presented as a percentage. We used the Cochran test to compare standard nomograms. Receiver operating characteristic (ROC) curve analysis was used to evaluate the predictive value of the ARr for standard nomograms (Gautier et al., Pettersen et al., Cantinotti et al.) and to determine its best cut-off value, sensitivity, and specificity. Moreover, the positive predictive value (PPV), negative predictive value (NPV) and accuracy of the ARr were calculated. A *P* value < 0.05 was considered statistically significant. Data were analyzed using SPSS software (v.21, IBM).

## Results

### Aortic Root Diameter as Expressed by the z-Score

Among the 190 patients who qualified for the study, aortic root dilation as expressed by the *z*-score was diagnosed in 54 (28.42%) patients using Gautier et al.’s nomogram, in 57 (30%) patients using Pettersen et al.’s nomogram, and in 60 (31.58%) patients using Cantinotti et al.’s nomogram (Table 4). In a detailed analysis we found that in 11 (5.79%) patients, the final results (dilated or non-dilated aortic root) that were obtained using different *z*-score formulas were inconsistent (Table 5). This discordance was significant between formulas based on nomograms from Gautier et al. and Cantinotti et al. (*P* = 0.034). Such discordances between the other nomograms were not significant, including Gautier et al. vs Pettersen et al. (*P* = 0.317) and Pettersen et al. vs Cantinotti et al. (*P* = 0.180). Similar discordance between nomograms was observed in analysis of the mean aortic root diameter expressed as a *z*-score for the whole study population (Table 4). There was a significant difference between Gautier et al.’s and Cantinotti et al.’s nomograms (*P* = 0.041), while there were no significant differences between the other nomograms (Gautier et al. vs Pettersen et al., *P* = 0.057 and Pettersen et al. vs Cantinotti et al., *P* = 0.887).

**Table 4:** Analysis of the aortic root diameter according to different *z*-score nomograms

Applied nomograms	Aortic root diameter as expressed by the *z*-score, range (*n* = 190)	Aortic root diameter as expressed by the *z*-score, mean ± SD (*n* = 190)	Aortic root dilation, *n* (%)
Gautier et al.	− 3.17 to 7.14	0.89 ± 1.99	54 (28.42)
Pettersen et al.	− 1.75 to 6.17	1.27 ± 1.64	57 (30.00)
Cantinotti et al.	− 2.66 to 7.2	1.30 ± 2.12	60 (31.58)

**Table 5:** Detailed data of patients in whom results that were obtained from different on-line *z*-score calculators were inconsistent

Patient no	Age (years)	Sex (F/M)	Weight (kg)	Height (cm)	BSA	Aortic root diameter (mm), leading edge technique	Aortic root diameter (mm), inner edge technique	Aortic root diameter (*z*-score), Gautier et al	Aortic root diameter (*z*-score), Pettersen et al	Aortic root diameter (*z*-score), Cantinotti et al
25	16	M	73	194	2.03	38	38	*2.16*	1.90	*2.84*
52	7	M	23	123	0.89	26	25	*2.14*	1.95	*2.48*
70	17	F	60	178	1.75	34	33	1.99	*2.10*	*2.03*
73	13	M	40	164	1.38	30	30	1.45	*2.24*	*2.31*
87	0.25	F	11	82	0.49	20	18	*2.37*	1.92	1.82
92	11	F	47	172	1.54	31	31.5	1.60	*2.32*	*2.29*
95	17	M	59	183	1.78	33	33	1.60	*2.01*	*2.02*
99	8	F	30	156	1.19	28.5	27	1.94	1.69	*2.07*
136	13	M	39	160	1.35	29	29	1.20	1.98	*2.06*
140	11	M	48	162	1.49	30	30	1.06	*2.00*	1.82
162	15	M	58	183	1.76	35	33	1.78	*2.06*	*2.07*

The values of a *z*-score ≥ 2 (dilated aortic root) are marked in italics

*F* female, *M* male

### ARr

The ARr was calculated for all 190 patients. In each patient, the ARr was calculated using aortic root measurements obtained by two different techniques, as described above. The ARr ranged from 12.3 to 35.6, with a mean value of 18.09 ± 3.90 using the leading edge technique, and ranged from 12.3 to 34.2, with a mean value of 17.78 ± 3.85 for the inner edge technique (Table 6).

**Table 6:** ARr as calculated using aortic root measurements obtained by two different techniques (leading edge, end-diastole and inner edge, mid-systole)

	Range	Mean value ± SD
ARr (leading edge, end-diastole)	12.3–35.6	18.09 ± 3.90
ARr (inner edge, mid-systole)	12.3–34.2	17.78 ± 3.85

**Fig. 2:**
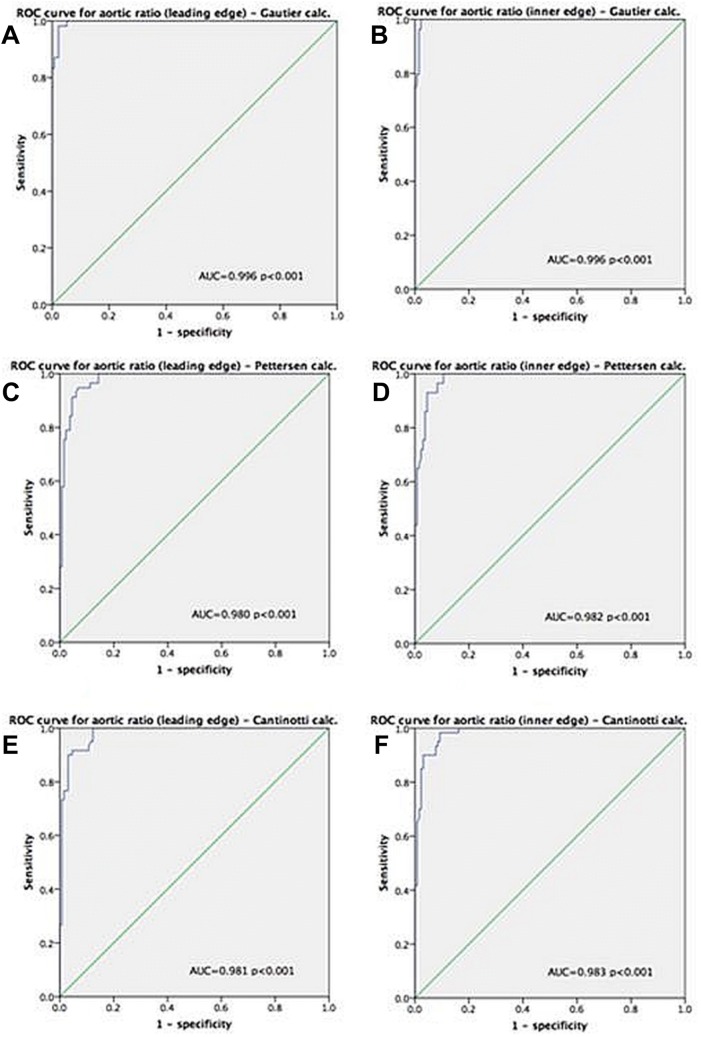
ROC curves for identifying the optimal ARr cut-off point for differentiation of a dilated or non-dilated aortic root. **a** ARr as calculated by the leading edge method compared with the Gautier et al. nomogram. **b** ARr as calculated by the inner edge method compared with the Gautier et al. nomogram. **c** ARr as calculated by the leading edge method compared with the Pettersen et al. nomogram. **d** ARr as calculated by the inner edge method compared with the Pettersen et al. nomogram. **e** ARr as calculated by the leading edge method compared with the Cantinotti et al. nomogram. **f** ARr as calculated by the inner edge method compared with the Cantinotti et al. nomogram

Identification of the optimal ARr cut-off point for differentiation of a dilated or non-dilated aortic root was performed using ROC curve analysis. The calculated optimal cut-off value for aortic root dilation was ≥ 18.7 (Fig. 2a–f). At this cut-off point, sensitivity of the ARr ranged from 88.3% to 100% (depending on the nomogram used) and specificity ranged from 94% to 97.8% (Table 7). Similar good results were obtained for ARr calculated using two different techniques of aortic root diameter measurement. ROC curve analysis showed that the area under the curve for predicting aortic root dilation was between 0.980 and 0.996 depending on the nomogram used for comparison (Fig. 2a–f).

**Table 7:** Sensitivity and specificity of the ARr in identifying aortic root dilation at the cut-off point of 18.7 in relation to the results obtained using three *z*-score formulas

	Gautier et al	Pettersen et al	Cantinotti et al
ARr (leading edge, end-diastole)	Sensitivity: 100% Specificity: 94.9%	Sensitivity: 93% Specificity: 94%	Sensitivity: 91.7% Specificity: 95.4%
ARr (inner edge, mid-systole)	Sensitivity: 100% Specificity: 97.8%	Sensitivity: 89.5% Specificity: 95.5%	Sensitivity: 88.3% Specificity: 96.9%

Individuals in whom the ARr failed to identify aortic root dilation were the subject of further assessment (Table 8). In five patients, the ARr was inconsistent with the results obtained using Pettersen et al.’s and Cantinotti et al.’s nomograms, but consistent with Gautier et al.’s nomogram. In one patient, the ARr was inconsistent with the results obtained using Pettersen et al.’s nomogram, but consistent with that using Gautier et al.’s and Cantinotti et al.’s nomograms. Finally, in two patients, the ARr was inconsistent with results obtained from Cantinotti et al.’s nomogram, but consistent with Gautier et al.’s and Pettersen et al.’s nomograms. In conclusion, in all of the patients in whom the ARr failed to identify aortic root dilation, the results (dilated or non-dilated) were inconsistent using different nomograms.

**Table 8:** Detailed data of patients in whom the ARr failed to identify aortic root dilation

Patient no	Age (years)	Sex (M/F)	Weight (kg)	Height (cm)	BSA	Aortic root diameter (*z*-score), Gautier et al	Aortic root diameter (*z*-score), Pettersen et al	Aortic root diameter (*z*-score), Cantinotti et al	ARr (leading edge, end-diastole)	ARr (inner edge, mid-systole)	Comment
73	13	M	40	164	1.38	1.45	*2.24*	*2.31*	18.30	18.30	The ARr was inconsistent with results from Pettersen et al.’s and Cantinotti et al.’s nomograms, but consistent with Gautier et al.’s nomogram
92	11	F	47	172	1.54	1.60	*2.32*	*2.29*	18.0	18.3	
95	17	M	59	183	1.78	1.60	*2.01*	*2.02*	18.0	18.0	
70	17	F	60	178	1.75	1.99	*2.10*	*2.03*	19.1	18.5	
162	15	M	58	183	1.76	1.78	*2.06*	*2.07*	19.1	18.0	
140	11	M	48	162	1.49	1.06	*2.00*	1.82	18.5	18.5	The ARr was inconsistent with results from Pettersen et al.’s nomogram, but consistent with Gautier et al.’s and Cantinotti et al.’s nomograms
99	8	F	30	156	1.19	1.94	1.69	*2.07*	18.3	17.3	The ARr was inconsistent with results from Cantinotti et al.’s nomogram, but consistent with Gautier et al.’s and Pettersen et al.’s nomograms
136	13	M	39	160	1.35	1.20	1.98	*2.06*	18.1	18.1	

The values of a *z*-score ≥ 2 (dilated) are marked in italics and values < 2 (non-dilated) are marked in underlined

As mentioned above, there were discrepancies between the results of the *z*-scores obtained using different nomograms, and therefore, there was lower sensitivity of the ARr at the cut-off point of ≥ 18.7. Consequently, in accordance with ROC curves, ARr cut-off points were selected at which a sensitivity of 100% would be achieved. These additional analyses were performed for *z*-score calculations on the basis of Pettersen et al.’s and Cantinotti et al.’s nomograms because the *z*-score calculations based on Gautier et al.’s nomogram had 100% sensitivity at the cut-off point of ≥ 18.7. We found that to acquire a sensitivity of 100% for both nomograms the cut-off point should be set at ≥ 18.0 (Table 9).

**Table 9:** Cut-off points for the ARr at which a sensitivity of 100% was achieved

	Gautier et al.	Pettersen et al.	Cantinotti et al.
Cut-off point at which the sensitivity was 100% (for the ARr, leading edge)	18.7	18.0	18.0
Cut-off point at which the sensitivity was 100% (for the ARr, inner edge)	18.7	18.1	18.0

We then calculated the PPV, NPV, and accuracy of the ARr in identifying aortic root dilation at the cut-off points of 18.7 and 18.0. These results confirmed the high quality of the ARr (Tables 10, 11).

**Table 10:** PPV, NPV, and accuracy of the ARr in identifying aortic root dilation at the cut-off point of ≥ 18.7 in relation to the results of the *z*-score obtained using three standard nomograms

	Gautier et al.	Pettersen et al.	Cantinotti et al.
ARr (leading edge, end-diastole)	PPV: 88.5% NPV: 100% Accuracy: 96.3%	PPV: 86.9% NPV: 96.9% Accuracy: 93.7%	PPV: 90.2% NPV: 96.1% Accuracy: 94.2%
ARr (inner edge, mid-systole)	PPV: 94.7% NPV:100% Accuracy: 98.4%	PPV: 89.5% NPV: 95.5% Accuracy: 93.7%	PPV: 93% NPV: 94.7% Accuracy: 94.2%

## Discussion

The primary role of pediatric cardiologists who suspect or diagnose a genetically determined connective tissue disease (e.g., MFS, EDS, Loeys–Dietz syndrome, and others) is to assess whether the aortic root is dilated. This is not only crucial for confirmation of diagnosis, but also for strategic planning of further cardiac care of the patient (including additional imaging studies, rate of follow-up assessment, and implementation of pharmacological therapy), as well as qualification for sport.

**Table 11:** PPV, NPV, and accuracy of the ARr in identifying aortic root dilation at the cut-off point of ≥ 18.0 in relation to the results of the *z*-score obtained using three standard nomograms

	Gautier et al.	Pettersen et al.	Cantinotti et al.
ARr (leading edge, end-diastole)	PPV: 71.1% NPV: 100% Accuracy: 88.4%	PPV: 75% NPV: 100% Accuracy: 90%	PPV: 78.9% NPV: 100% Accuracy: 91.6%
ARr (inner edge, mid-systole)	PPV: 76.1% NPV:100% Accuracy: 91.1%	PPV: 80.3% NPV: 100% Accuracy: 92.6%	PPV: 83.1% NPV: 99.2% Accuracy: 93.2%

As already mentioned in the Introduction, the aortic root diameter depends on the patient’s age, sex, and BSA. Therefore, to determine whether an aortic root is dilated, a dedicated *z*-score calculation tool should be used. However, the use of on-line *z*-score calculators is cumbersome and time-consuming for physicians who use them infrequently. Screening adults for aortic root dilation is much easier. Although the diameter of the aortic root in adults is affected by the factors mentioned above, there are approximate norms that can be applied (36 mm in women, 40 mm in men) [12]. Consequently, in adults, only patients with an aortic root diameter close to the upper limit have to be verified with *z*-score calculations. However, the great diversity in age and BSA in the pediatric population has made establishing a single or approximate norm for the aortic root diameter impossible. Pediatric cardiologists can only use the visual proportions of the aortic root to its neighboring heart structures, such as the left atrium. However, such an assessment is largely approximate and only allows for confirmation of large aortic root dilation.

Therefore, there is a strong rationale to create a simple screening tool for identifying aortic root dilation in the pediatric population. Consequently, we examined the ARr by analyzing a child population with a wide age range (3 months–18 years), with suspicion or diagnosis of connective tissue disease. This new tool corrects the aortic root diameter purely on the basis of a patient’s height because body mass does not significantly affect the aortic root diameter [13]. Notably, this is why thin or obese patients may be mistakenly classified if *z*-score formulas including BSA are applied.

The ARr, which is a simple ratio of the aortic root diameter to a patient’s height, does not require access to any nomograms or on-line *z*-score calculators. This tool allows for rapid and precise assessment of whether the aortic root is dilated. Notably, a simple calculation can be performed at the bedside using a basic electronic calculator (e.g., a smartphone). To calculate the ARr, the diameter of the aortic root (in mm) is divided by the patient’s height (in cm). This unit inconsistency with regard to ARr components was deliberate. Routinely, height is expressed in centimeters and the aortic root diameter in millimeters. Therefore, performing calculations without additional unit conversion is easy. Finally, multiplying the obtained figure by 100 enables easier assimilation of the ratio, and thereby avoiding cumbersome fractions. We determined the sensitivity and specificity of the ARr for assessing aortic root dilation by comparing it with the results achieved using the three most widely used nomograms. Surprisingly, we demonstrated significant inconsistencies in aortic root diameter expressed as a *z*-score, which was calculated using three different *z*-score calculators. In fact, in our population, as many as 5.79% of patients were classified differently (dilated or non-dilated aortic root) by various nomograms. Consequently, the lack of fully reliable benchmarks hindered defining the most optimal (i.e., the highest possible sensitivity and specificity) cut-off point for ARr. However, based on our own experience with the above-mentioned nomograms and taking into account previous reports, which indicated that some nomograms over-diagnose aortic root dilation, our primary calculation referred to Gautier et al.’s nomogram [15, 18]. The cut-off point of ≥ 18.7 allowed us to obtain 100% sensitivity and high specificity (94.9%–97.8%). Such a cut-off point also guaranteed a high, but not 100%, sensitivity and specificity in relation to the other two analyzed nomograms (Table 4). This cut-off point appears to be satisfactory because the values of the area under the curve, PPV, NPV, and accuracy of the ARr at this cut-off point were high. However, because of a lack of sufficient evidence of the advantage of the Gautier nomogram over the other tests, we estimated the cut-off point at which 100% sensitivity would be achieved for all nomograms. We found that the cut-off point was ≥ 18.00. Therefore, we recommend using this limit because it is the safest and it allows for detection of all patients with aortic root dilation, although at the cost of a lower specificity (Table 9).

To the best of our knowledge, the ARr is the second proposed tool that is dedicated to screening for aortic root dilation in pediatric patients with the suspicion or established diagnosis of MFS and Marfan-like disorders. In a previous study, the ratio of the aortic root to the descending aorta diameter was proposed [19]. In this previous study, the ratio was verified in a population of 35 patients with MFS and 52 normal controls. At the cut-off point of ≥ 2, the authors achieved 100% sensitivity, but specificity was only 58% [19]. We performed validation of the tool designed by Kemna et al. in our population and obtained 91–92% sensitivity and 78–81% specificity (depending on the *z*-score calculator used for comparison). These results indicate that the ratio proposed by Kemna et al. is not suitable for precise screening of aortic root dilation and certainly cannot be used in patients with dilation of the descending aorta. As we previously demonstrated, the latter does not occur as often as dilation of the aortic root, but it is still one of the most characteristic abnormalities in patients with MFS and Marfan-like disorders [20].

## Limitations

Further studies in a larger population are required to externally validate the ARr. Because of the fact that the ARr was designed on the basis of analysis of a population with the suspicion or diagnosis of a genetically determined connective tissue disease, the majority of patients were tall and slim. Therefore, the question may arise whether this screening tool is applicable to the general pediatric population. Importantly, the great diversity in percentile height range (2–99.9, mean ± SD: 78.80 ± 25.30) and weight (0.1–99.9, mean ± SD: 44.78 ± 28.82) of the study participants indicated significant group heterogeneity in physique (Table 2). Therefore, we believe that the ARr may also be a reliable screening tool in the general child population. Finally, the analyzed population age span was wide (3 months–18 years). Therefore, the ARr is generally applicable in all children. Nevertheless, the youngest age group (3 months–3 years) was relatively sparsely represented, causing the tool to be less plausible in this age group. Finally, all patients included in the study were Caucasian, which may potentially hamper application of the ARr in patients of other ethnicities.

## Conclusion

The ARr, which is a simple quotient of the aortic root diameter to the patient’s height, is a useful screening test for aortic root dilation in the pediatric population. The ARr allows for rapid and precise screening for aortic root dilation and, unlike the classic analysis, it does not necessitate access to any nomograms or on-line calculators. The ARr was designed as a screening tool for identifying aortic root dilation in binary classification. Those in whom the ARr is positive for dilation require further assessment with *z*-score calculation tools to determine its severity. The ARr is suitable for children of all ages, and even though it is being initially created for patients with connective tissue diseases, it can probably be used in the entire child population. Undeniably, the largest advantage of the ARr is its efficiency. Even when taking into consideration the large discrepancies in the results between *z*-score calculators, 100% sensitivity can still be achieved.

## Abbreviations

ARr: Aortic root ratio
EDS: Ehlers–Danlos syndrome
MFS: Marfan syndrome
NPV: Negative predictive value
PPV: Positive predictive value
ROC curve: Receiver operating characteristic curve
